# Harvesting the Mouse Genome

**DOI:** 10.1002/cfg.198

**Published:** 2002-08

**Authors:** Marc Botcherby

**Affiliations:** MRC UK HGMP Resource Centre Genome Campus Hinxton, Cambridge CB10 1SB UK

## Abstract

The sequencing of the black 6 mouse (strain C57Bl/6) has reached an important juncture. The BAC fingerprint map is almost complete, the BACs have been endsequenced and a seven-fold coverage whole-genome shotgun has been assembled. Now the BAC-by-BAC sequencing phase is under way and in-depth comparative analysis can be carried out on regions that have been the subject of targeted sequencing. This paper reviews the progress so far and looks forward to the promises of finished sequence.


**Comparative and Functional Genomics**
*Comp Funct Genom* 2002; **3**: 319–324.
Published online in Wiley InterScience (www.interscience.wiley.com). **DOI:** 10.1002/cfg.198

**Review**

### Harvesting the mouse genome

Marc Botcherby*
*MRC UK HGMP Resource Centre, Genome Campus, Hinxton, Cambridge CB10 1SB, UK*

**Correspondence to:*
*Marc Botcherby, MRC UK HGMP Resource Centre, Genome Campus, Hinxton, Cambridge CB10 1SB, UK.*
*E-mail: mrbotche@hgmp.mrc.ac.uk*

Received: 6 June 2002
Accepted: 17 June 2002

### Abstract

**The sequencing of the black 6 mouse (strain C57Bl/6) has reached an important juncture. The BAC fingerprint map is almost complete, the BACs have been end-sequenced and a seven-fold coverage whole-genome shotgun has been assembled. Now the BAC-by-BAC sequencing phase is under way and in-depth comparative analysis can be carried out on regions that have been the subject of targeted sequencing. This paper reviews the progress so far and looks forward to the promises of finished sequence. Copyright © 2002 John Wiley & Sons, Ltd.**

**Keywords:** **mouse; sequencing; whole genome shotgun; ARACHNE; cDNA**

### Introduction

Lessons have been learnt from the Human Genome Project, which the genome centres have been able to apply to the mouse. The resources of the large genome centres have been coordinated in order to create an integrated strategy to the mouse sequencing project. Mapping, end-sequencing and whole genome shotgun resources have been obtained to support the final clone-based genomic sequence.

As the public draft of the mouse genome was recently announced by the International Mouse Sequencing Consortium and the mouse sequencing milestones have been brought forward, it is worth looking at the various large-scale on-going projects in the world of mouse genomics.

### Status of the mouse genome map

A number of internationally available mapping resources facilitate the work of mouse genome researchers. The reference libraries for mouse genomic studies, RPCI 23 and RPCI 24 C57Bl/6 Bacterial Artificial Chromosomes (BACs), have been mapped by fingerprinting and end sequencing. Approximately 450 000 BACs were end-sequenced at the Institute for Genome Research (TIGR) (available from the TIGR home page and mirrored at the HGMP-RC). Around 306 000 BACs were fingerprinted at the British Columbia Genome Sequencing Center [viewable using the internet Contig Explorer (iCE; mouse database); the BAC tiling path selected for sequencing is also available from the Ensembl mouse page, using the 'contig view' option]. These were brought together in an assembly which was supported by 41 000 homology matches to the human sequence, and 16 997 mouse mapping markers provided by previous genetic and radiation hybrid maps. The average contiguous assembly (contig) length is 9.3 Mb and over 2.6 Gb (90%) of the mouse map is aligned to the human sequence. It is made available by the Ensembl project on their mouse page.

### Status of the public mouse genome sequence

41 million whole genome shotgun (WGS) reads from the C57bl/6 strain, representing a seven-fold coverage of the genome, were carried out by the Mouse Genome Sequencing Consortium. The data are available from the Ensembl trace repository, which can be searched using the SSAHA search engine. These reads have been assembled at the Sanger Institute, using the PHUSION assembly

Copyright © 2002 John Wiley & Sons, Ltd.

engine (Z. Ning and J. Mullikin, unpublished data) and at the Whitehead Institute using the ARACHNE assembler [1], using mapping information as support. In-depth comparisons of the two assemblies found them to be comparable by many criteria; however, the ARACHNE assembly was eventually selected as the basis for further analysis by the Mouse Genome Sequencing Consortium and is available from Ensembl. The N50 contig length value is 25 kb, which means that there is a 50% probability that a given base will find itself in a contig of 25 kb or greater. The N50 scaffold length value is 17 Mb. A contig is defined as a contiguous assembly of sequencing reads, whereas a scaffold is a structure in which a number of contigs are linked together and ordered on the basis of paired sequences of genomic clones. Ultracontigs were then constructed by bringing scaffolds together on the basis of the BAC physical map.

An illustration of how the emerging sequence is aligned to the physical map is shown in the two Mouse Ensembl screenshots in Figure 1. Figure 1A represents the WAGR region on mouse chromosome 2, which is fully sequenced. The display shows the genomic sequence features aligned to the BACs mapping to the region. The clones can be obtained from CHORI or a resource centre such as the HGMP-RC (MRC Geneservice) for further analysis, such as functional research. The region featured in Figure 1B is, as yet, more thinly sequenced, illustrating the variation in coverage that exists at this time.

### The Celera draft sequence

Another whole genome shotgun project was carried out by the Celera corporation. This mouse genome assembly comprises approximately 40 million reads. These reads are taken from a mixture of strains; 2.3-fold coverage is from the A/J strain, 1.6 from the DBA/2J strain, and 1.1 from the 129X1/SvJ strain. The assembly incorporates 1.7-fold coverage from the publicly available C57Bl/6 sequence carried out by the International Mouse Sequencing consortium. Additional reads from another strain, 129X1/SvnJ, bolster-up the assembly, which represents a 7.8-fold coverage of the genome. The average contiguous assembly is 27.6 kb and the average scaffold length is 33 Mb, with the largest being 112 Mb in length. In this assembly, 100 scaffolds represent 90% of the genome.

Because data from different strains is included, the assembly is a good resource for single nucleotide polymorphism (SNP) discovery. Although the figures are not adjusted for sequencing errors, 3.4 million SNPs have been mapped; 40% of these SNPs are intragenic, of which 91% have been validated experimentally. This resource is available on subscription; details are available from Celera.

### Completing the public mouse genome sequence

The Mouse sequencing project will only be considered fully completed when the tiling path of mapped genomic clones from the RPCI 23 and 24 reference libraries have been fully sequenced and finished to the internationally accepted standards. The availability of the whole genome shotgun assembly will allow the sequencing centres involved to adopt a hybrid strategy, in which the available WGS sequence will be included in the assembly and finishing of BACs.

Some centres will be bolstering their assembly with the appropriate ARACHNE contigs and quality values, using tools such as FetchWGS (M. Griffiths, unpublished data), and others retrieving the original reads which made up the contig from the trace repository. Whichever strategy is employed, the DNA sequence production teams will need to provide fewer reads and the sequencing centres are currently assessing the saving in production reads required. Not all have come to a conclusion, but work carried out at the Sanger Institute indicates a figure of 1100 reads per 100 kb of genomic sequence, as reported by Lucy Mathews and colleagues at the Cold Spring Harbor Genome Sequencing and Biology conference last May. This represents a reduction of around 50% on the depth

**Figure 1.** Screenshots of mouse genome coverage from Ensembl. (A) A region that has been fully sequenced, showing genomic features as well as sequenced BACs mapping to the region (six finished BACs now span this region). (B) A region that is, as yet, only covered by whole genome shotgun assembly

Copyright © 2002 John Wiley & Sons, Ltd.

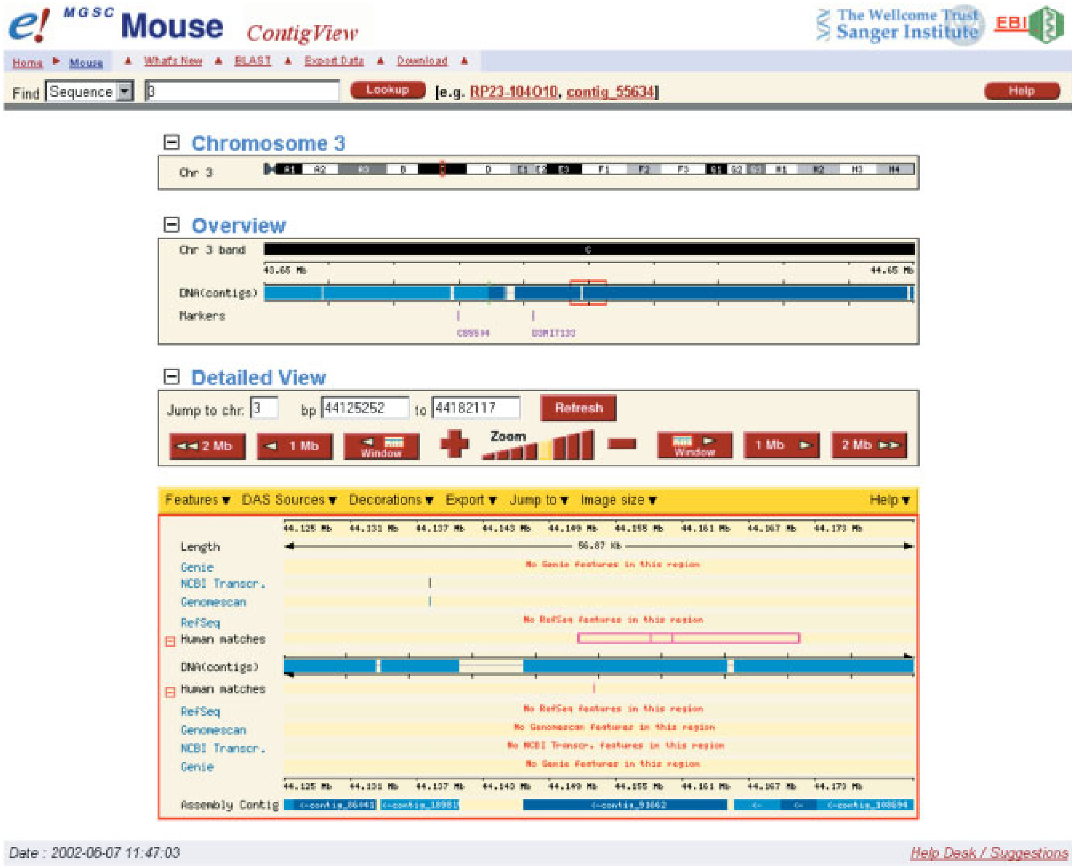

**Figure 1.** Continued of coverage required before the assembled whole-genome shotgun data became available.

### Targeted sequencing

In addition to the general approach taken to the sequencing of the mouse genome, some regions have been selected for fast-track finished sequence, within the coordinated strategy, sequencing BACs from the same libraries as the mouse sequencing project and entering them in the NIH clone registry, in order to avoid duplication of effort, e.g. the American National Human Genome Research Institute (NHGRI) will accept requests for sequencing individual BACs of high biological interest, the Joint Genome Institute (JGI) is sequencing regions syntenic with human chromosome 19, and the UK mouse sequencing consortium is targeting regions that are the focus of intensive study.

### UK mouse sequencing consortium

In view of the ‘patchy’ nature of the sequence coverage (see Figure 1), in order to support on-going, detailed, biologically-based projects, some regions have been targeted for priority sequencing, and this is the approach taken by the Medical Research Council. A UK Mouse Sequencing Consortium was set up in 1999 with the brief of obtaining the finished sequence for regions of particular interest to the British mouse genomic community ahead of the rest of the genome, by October 2002. The regions have been selected because they form the basis of on-going projects, and all are the target of mutation screens within the ENU mutagenesis project. Briefly, the four regions are a 9 Mb region on mouse chromosome 2 (human 11p13–14) containing the WAGR region, as well as other known mutations, a 22 Mb region on mouse chromosome 4 (human 9p23 and 9q33) around the tyrp1 (brown) locus, the

Copyright © 2002 John Wiley & Sons, Ltd. 

Del(13)Svea36H deletion region on mouse chromosome 13, covering 14 Mb, and the *Dmd* to *Ar* region of mouse chromosome X.

In addition to these four main regions, which amount to 50 Mb, applications to sequence additional small regions were invited from the UK scientific community. 46 BACs covering 19 loci originating from 17 collaborators were accepted for sequencing within the scope of these additional projects, and most are finished with the rest in shotgun phase. The regions on chromosomes 2 and 4 are on target to be completed by the end of the project and the regions on 13 and X are likely to be finished ahead of the end of the project. The chromosome 13 region represents 14 Mb of contiguous sequence and therefore provides the opportunity for the longest stretch to date of contiguous sequence comparison between species.

### Comparative studies

Furthermore, the close analysis of these sequence comparisons provides important information in the form of conserved regulatory elements, e.g. the finished sequence for the WAGR region from WT1 to ELP4 is available for human, mouse and *Fugu rubripes* [4]. The areas of conservation of these three genomic sequences are aligned to the

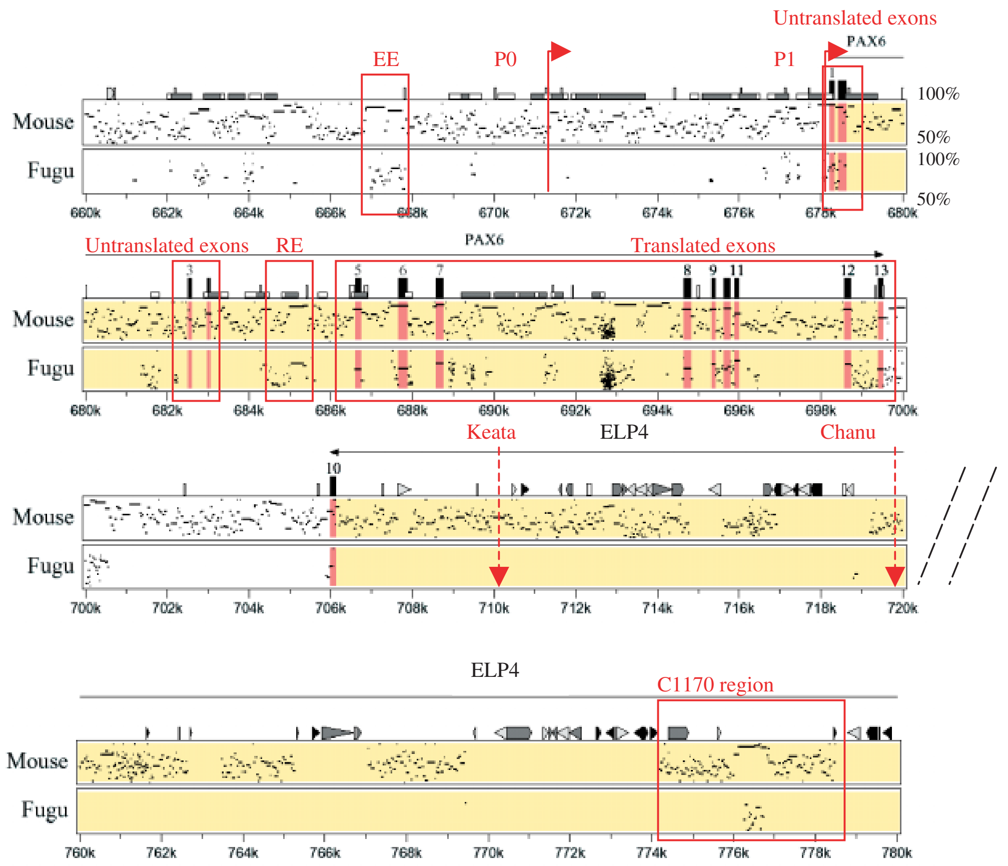

**Figure 2.** A percentage identity plot (PIP) of the PAX6 locus. Part of a large-scale comparison of a 1 Mb human region to the equivalent mouse and *Fugu* sequence. The top line represents the human annotation: gene name with orientation indicated by arrow, and exons shown as numbered black boxes. Repeats are shown by shaded and clear arrows and CpG islands are shown as narrow boxes. In each panel, the top plot represents the percentage identity to the mouse sequence, and the lower plot, the percentage identity to the *Fugu* sequence. The *x* axis is the sequence length in kilobases and the *y* axis the percentage identity. Red boxes highlight conserved features in the sequence, such as the ectodermal (EE) and intron 4 retinal enhancer (RE), the untranslated and translated exons of PAX6, and the C1170 control region. The promoters, P0 and P1 are indicated, as are the positions of known chromosomal rearrangements that abolish PAX6 gene expression

Copyright © 2002 John Wiley & Sons, Ltd. 

genomic features of the human sequence in a percentage identity plot (PIP) [5] (Figure 2). Analysis of these sequences by Veronica Van Heyningen and colleagues reveals candidates for regulatory elements, some of which have been confirmed in animal experiments [3], e.g. the Ectodermal Enhancer and the Retinal Enhancer are clearly conserved in mouse and *Fugu*, as are the promoters and the C1170 Box 123 *cis*-regulatory region [2]. Note also that the untranslated exons 3 and 4 are not conserved in *Fugu*, whereas they are in mouse. The translated exons 5–13 are conserved in all three species.

### Mouse cDNA sequencing

As the focus starts to shift from the genomic to the post-genomic era, efforts are being made to obtain finished sequence of full-length cDNA libraries. The RIKEN Institute, in Yokohama City, Japan, has just organized the annotation of the first 60 000 clones from the FANTOM 2 project. The average length of cDNA was 1974 bp, the longest 12 349 bp. Although a publication is not out yet, an overview of the results was presented in poster form by Yasushi Okazaki and colleagues at the Genome Sequencing and Biology meeting at the Cold Spring Harbor Laboratories, in May.

60 770 full-length cDNA sequences were aligned to the mouse genome. Of these, 36 000 loci were annotated, with 47 000 variants, approximately 20% matched unknown ESTs and 20% were unclassifiable. A closer study of 7000 pairs of sense and antisense genes was also carried out.

### Conclusion

The availability of the mouse genomic resources, particularly that of the whole-genome shotgun data, will have a marked effect on the endpoints of the mouse genome sequencing project, bringing the target dates forward. It is now estimated that BACs will be in 'deep-shotgun' phase (most of the sequence data being obtained) by next spring, with the likely date for a finished mouse genome sequence now being 2005.

### Useful websites

**http://www.ensembl.org/Mus_musculus** (annotated view of the mouse genome)

**http://trace.ensembl.org** (trace repository for a range of projects, including Mouse Sequencing Consortium)

**http://icebox.bcgsc.ca/ice/mouse.html** (iCE-internet Contig Explorer - Mouse Database)

**http://mrcseq.har.mrc.ac.uk** (MRC UK Mouse Sequencing Consortium)

**http://www.tigr.org/tdb/bac_ends/mouse/bac_end_intro.html** (TIGR BAC end sequences)

**http://www.hgmp.mrc.ac.uk/** (MRC UK HGMP Resource Centre)

**http://www.chori.org/bacpac/** (CHORI BAC-PAC resources)

**http://www.celera.com** (Celera)

**http://www.gsc.riken.go.jp/e/FANTOM/** (FANTOM annotation project)

**http://bio.cse.psu.edu/pipmaker/** (PIPmaker)

### References

1. Batzoglou S, Jaffe DB, Stanley K, *et al.* 2002. ARACHNE: a whole-genome shotgun assembler. *Genome Res* **12**: 177–189.
2. Griffin C, Kleinjan DA, Doe B, van Heyningen V. 2002. New 3prime prime or minute elements control Pax6 expression in the developing pretectum, neural retina and olfactory region. *Mechan Dev* **112**: 89–100.
3. Kleinjan DA, Seawright A, Elgar G, van Heyningen V. 2002. Characterization of a novel gene adjacent to PAX6, revealing synteny conservation with functional significance. *Mammal Genome* **13**: 102–107.
4. Miles C, Elgar G, Coles E, Kleinjan DJ, Heyningen V, Hastie N. 1998. Complete sequencing of the *Fugu* WAGR region from WT1 to PAX6: dramatic compaction and conservation of synteny with human chromosome 11p13. *Proc Natl Acad Sci USA* **95**: 13 068–13 072.
5. Schwartz S, Zhang Z, Frazer KA, *et al.* 2000. PipMaker — a web server for aligning two genomic DNA sequences. *Genome Res* **10**: 577–586.

Copyright © 2002 John Wiley & Sons, Ltd. 
